# Exploring Fever of Unknown Origin Intelligent Diagnosis Based on Clinical Data: Model Development and Validation

**DOI:** 10.2196/24375

**Published:** 2020-11-30

**Authors:** Huizhen Jiang, Yuanjie Li, Xuejun Zeng, Na Xu, Congpu Zhao, Jing Zhang, Weiguo Zhu

**Affiliations:** 1 Department of Information Center Peking Union Medical College Hospital Chinese Academy of Medical Sciences and Peking Union Medical College Beijing China; 2 Department of Primary Care and Family Medicine Peking Union Medical College Hospital Chinese Academy of Medical Sciences and Peking Union Medical College Beijing China

**Keywords:** fever of unknown origin, intelligent diagnosis, machine learning, BERT, fever, misdiagnosis

## Abstract

**Background:**

Fever of unknown origin (FUO) is a group of diseases with heterogeneous complex causes that are misdiagnosed or have delayed diagnoses. Previous studies have focused mainly on the statistical analysis and research of the cases. The treatments are very different for the different categories of FUO. Therefore, how to intelligently diagnose FUO into one category is worth studying.

**Objective:**

We aimed to fuse all of the medical data together to automatically predict the categories of the causes of FUO among patients using a machine learning method, which could help doctors diagnose FUO more accurately.

**Methods:**

In this paper, we innovatively and manually built the FUO intelligent diagnosis (FID) model to help clinicians predict the category of the cause and improve the manual diagnostic precision. First, we classified FUO cases into four categories (infections, immune diseases, tumors, and others) according to the large numbers of different causes and treatment methods. Then, we cleaned the basic information data and clinical laboratory results and structured the electronic medical record (EMR) data using the bidirectional encoder representations from transformers (BERT) model. Next, we extracted the features based on the structured sample data and trained the FID model using LightGBM.

**Results:**

Experiments were based on data from 2299 desensitized cases from Peking Union Medical College Hospital. From the extensive experiments, the precision of the FID model was 81.68% for top 1 classification diagnosis and 96.17% for top 2 classification diagnosis, which were superior to the precision of the comparative method.

**Conclusions:**

The FID model showed excellent performance in FUO diagnosis and thus would be a potentially useful tool for clinicians to enhance the precision of FUO diagnosis and reduce the rate of misdiagnosis.

## Introduction

Fever is one of the most common symptoms in medicine [1]. A febrile temperature may boost the immune system to fight disease [2]. Prolonged fevers are usually complex to diagnose [3]. A fever of unknown origin (FUO) has remained a challenging diagnostic problem in recent decades [4].

As there are more than 200 causes of FUO [3], isolating the cause of an FUO is a great challenge for clinicians. Thus, many clinicians have been drawn to FUO research [5]. In 1961, Petersdorf and Beeson [6] defined FUO. There are usually 3 characteristics: (1) prolonged fever for more than 3 weeks, (2) recurrent fever with a temperature higher than 38.3℃, and (3) undiagnosed fever after a 1-week inpatient investigation [6,7]. The definition has been revised over time with regard to the classification and the duration of fever to be diagnosed [8,9]. The classification of FUO has been hotly debated in previous studies [10,11]. Usually, the categories of causes are infections, immune diseases, and tumors [12,13], and their treatment methods are considerably different, including anti-infection medication, hormones, and chemotherapy, respectively. Therefore, if the cause of an FUO is diagnosed to one category, regardless of the disease causing the FUO, the treatment direction can basically be determined, which would be meaningful for doctors. Infection is the most common cause of FUO [14]. Chow and Robinson [15] found that for more than one-half of children with FUO, the FUO was caused by an infection. Knockaert et al [9] proposed that the wait-and-see strategy may be better for prolonged prognosis in adults. Therefore, FUO is worth studying and exploring. Previous studies on FUO have mainly analyzed real patients' cases. de Kleijn and von der Meer [16] assessed 53 patients with FUO using a statistical analysis tool called PRISMA (Preferred Reporting Items for Systematic Reviews and Meta-Analyses) [17]. de Kleijn et al [18] analyzed 167 patients with FUO using fixed criteria. Similarly, Efstathiou et al [13] discriminated FUO into infectious and noninfectious causes. While these research results might explain how these patients with FUO were diagnosed, it was difficult to automatically determine the method because the limited amount of data sometimes caused overfitting. Little research on FUO has been done using machine learning methods.

In this paper, we proposed an FUO intelligent diagnosis (FID) model to classify the causes of FUO into 4 categories based on clinical data. Extensive experiments showed good performance of the model. In summary, we made the following contributions: (1) we innovatively introduced an FUO intelligent classification diagnosis model called FID, which can automatically group FUO cases into one of the categories of causes, (2) our experiments were based on real, desensitized data from Peking Union Medical College Hospital and thus, the cases were real and the results were more valuable and credible when applied to a clinical setting, and (3) we conducted extensive experiments to evaluate the performance of the FID based on the gradient boosting methods LightGBM and XGBoost, which performed better on small data sets; the FID model achieved better performance using LightGBM.

## Methods

### Modeling

**Figure 1:**
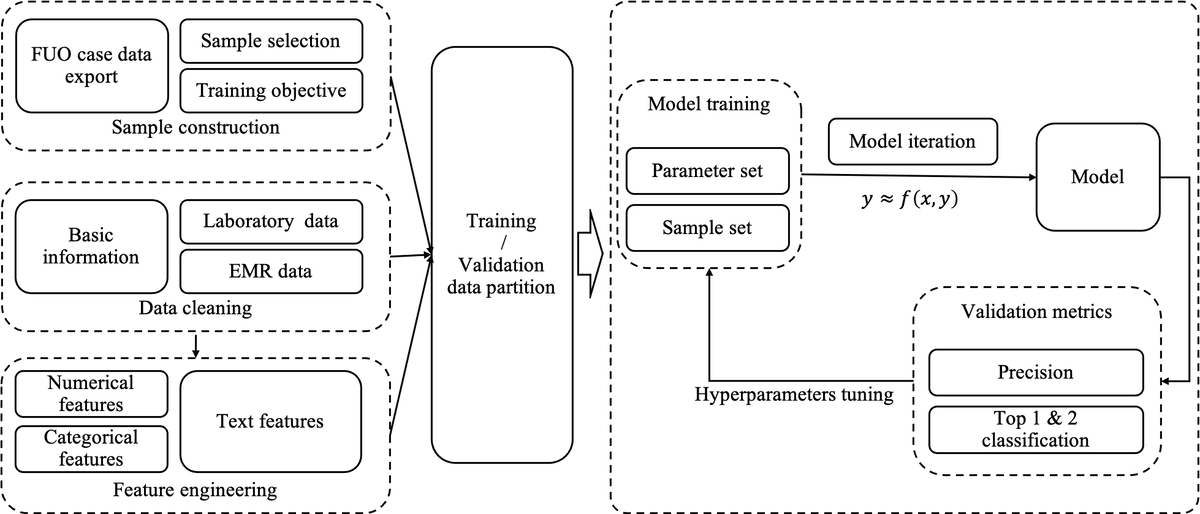
Structure of the fever of unknown origin (FUO) intelligent diagnosis (FID) model. The left side shows the sample construction, data cleaning, and feature engineering process; the right side shows the model training and tuning processes. EMR: electronic medical record.

In this paper, we proposed the FID model using LightGBM to intelligently diagnose patients with FUO into 1 of 4 causes using their basic information, clinical laboratory data, and electronic medical record (EMR) data. The structure of the FID model is shown in Figure 1. First, we classified the causes of FUO into 4 categories: infections, immune diseases, tumors, and others. Then, we cleaned the basic information and laboratory data and structured the EMR data via 2 methods: the bidirectional encoder representations from transformers (BERT) model [19] and Jieba [20]. Next, we extracted the features and trained the model through extensive experiments. Finally, by comparing the experimental results, we evaluated the performance of the FID model.

### Data Sources

**Figure 2:**
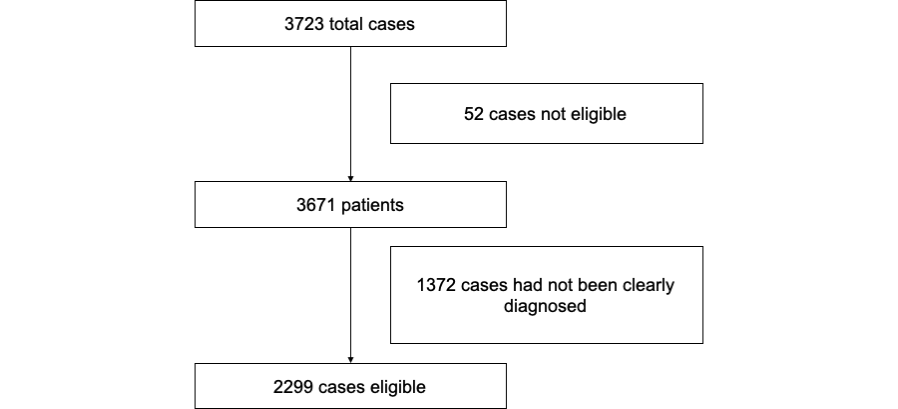
Data filtering process.

**Figure 3:**
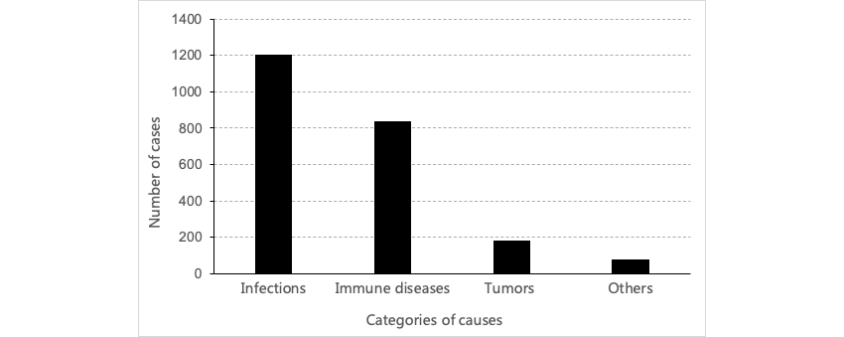
Distribution of the data set.

This research was based on data from 2299 desensitized FUO cases from Peking Union Medical College Hospital from June 1, 2012, to March 31, 2018, and the data filtering process is shown in Figure 2. The data contained basic information, laboratory results, and EMR data. One patient visit was taken as 1 sample; if a patient was admitted to the hospital twice, then the 2 visits were taken as 2 samples. There were 3723 total cases whose chief complaint included “fever.” First, we filtered out the 52 cases not eligible for FUO diagnosis, such as those with temperatures lower than 38.3°C. Then, we invited 3 doctors specializing in FUO diagnosis to help check the data and classifications. Two doctors divided the remaining cases into 4 categories: infections, immune diseases, tumors, and others. The third doctor checked the classifications, and if there were disagreements, the 3 doctors discussed the cases until they obtained a consistent classification. Based on the doctors’ suggestion, 1372 cases that had no confirmed diagnosis were filtered out. As a result, 2299 cases whose causes fit into 1 of the 4 categories remained for the experiments. Infections, immune diseases, tumors, and others accounted for 52.28% (1202/2299), 36.50% (839/2299), 7.83% (180/2299), and 3.39% (78/2299) of cases, respectively. The distribution of the data is shown in Figure 3. We randomly divided 80% of the data into the training set and the remaining 20% into the validation set, and we used the validation set data to evaluate the performance of the model.

### Model

#### Sample Structure

After obtaining the data marked by doctors, we needed to clean the basic information and laboratory data and obtain the structured EMR data. The EMR data were unstructured. To structure the EMR data, we used the BERT model. The BERT model was proposed by Devlin et al [19] in 2018, and it has greatly improved the text structuring process. We used the BERT-based pretrained model to process the text in this study. The input of BERT was all of the EMR text and the text in each line belonging to 1 patient. Each line in the output of BERT was a number vector of 768 dimensions. In addition, we compared the results based on BERT and Jieba. Using Jieba, we segmented all the text data and chose the top 100 text segmentations according to the counts of the words that occurred in all the text, such as “fever,” “infected,” and “lymph node.” In addition, stop words were filtered out manually. Then, for each of the 100 text segmentations, if it existed in the EMR text of 1 patient, we used 1 to represent the segmentation; otherwise, we used 0. Finally, the text of each patient was expressed by a vector of 100 dimensions.

#### Data Cleaning

The data were irregular after being extracted from the clinical system. Regarding the laboratory results, each item group consisted of too many cell items; therefore, laboratory data could be a thousand dimensions. However, there were some items that occurred only a few times, which were difficult for the model to learn. Therefore, we filtered out the items occurring less than 10 times. Finally, 214 items were left for the laboratory data. Then, we transformed all the values and units to be consistent. Regarding the synonyms, we fixed them to be the same. For example, “1 L” and “1000 ml” were fixed to “1000 mL.”

#### Feature Engineering

Feature engineering is the most important part of machine learning. The performance of a model is determined by feature engineering to a large extent. In this paper, there were 3 kinds of features: numerical features, categorical features, and text features.

Numerical features were objective data, such as age and heart rate. For the numerical features, we directly extracted the values as the features.

Categorical features were the classification indicators, such as gender and positive and negative symptoms. Categorical features were addressed using the LabelEncoder method [21] with numbers starting from 0, and then numbers were assigned to the features.

Text features, which could also contribute necessary information, were mainly from the EMR. They were structured in the previous step. Then, we merged the structured EMR features with all of the features. We used 2 methods to structure the text: using Jieba, the top 100 segmentations were selected as the features according to the counts of every word segment, and using BERT, all the text was structured as 768 features.

## Results

We used the LightGBM framework to train the model. LightGBM is a gradient boosting framework that is based on decision trees and has faster training efficiency, lower memory usage, and higher precision than other frameworks, as well as a large-scale data processing capability. During the training of LightGBM, there were many parameters that needed to be optimized, including the number of iterations, the learning rate, and the number of leaf nodes. During the experiments, we found that different parameters impacted the final results of the model to some extent. In this research, the parameters we used were a learning rate of 0.01 and number of leaves of 6 after the experiments. The output of the multiclassification model was the probability that each sample belonged to each category. Therefore, the classification with the largest prediction probability was generally regarded as the sample classification.

**Figure 4:**
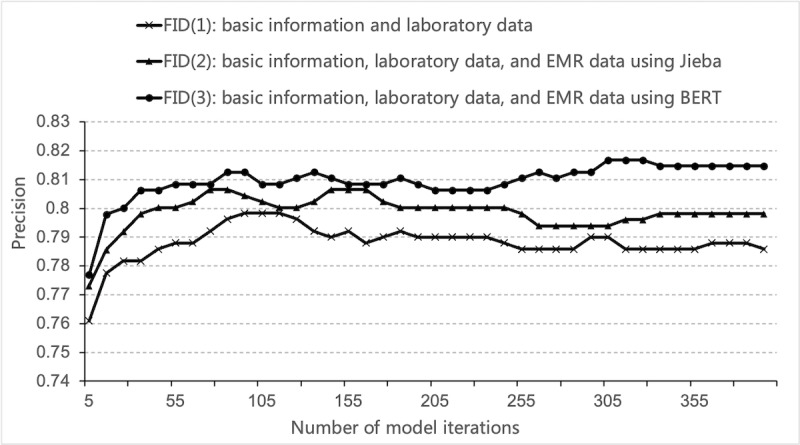
Performance of different data sets. FID: fever of unknown origin intelligent diagnosis. EMR: electronic medical record. BERT: bidirectional encoder representations from transformers.

Based on different features, we built 3 models: FID(1) was based on the basic information and laboratory features; FID(2) was based on the basic information, laboratory data, and EMR data using Jieba; and FID(3) was based on the basic information, laboratory data, and EMR data using BERT. The results are shown in Figure 4. The figure shows that the precision was further improved by increasing the number of features from the text data. After introducing the text features developed by BERT, we obtained the optimal model: FID(3). The precision was 81.68%. Experiments show that, in addition to structured data, unstructured text data also hides a lot of valuable information, which could improve the performance of the model.

**Figure 5:**
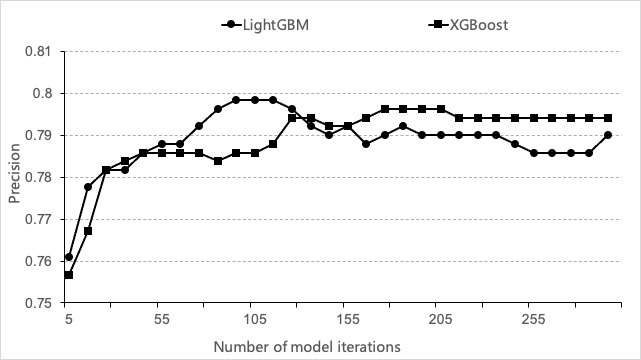
Performance based on the LightGBM and XGBoost algorithms. The abscissa represents the number of model iterations, and the ordinate shows the precision of the model.

In addition to the LightGBM algorithm, we also tested XGBoost, an algorithm with a similarly good performance as LightGBM. Both are gradient boosting algorithms. As shown in Figure 5, the precision of LightGBM was higher than that of XGBoost with relatively fewer iterations. When the number of iterations increased, the precision of XGBoost exceeded that of LightGBM, but it was still lower than the best performance of LightGBM. Therefore, we used LightGBM as the training algorithm for the subsequent experiments in this study.

**Figure 6:**
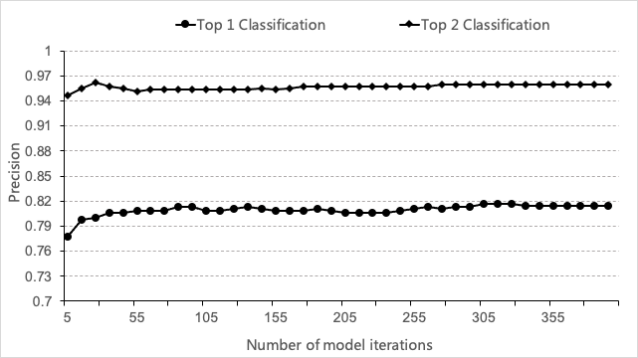
Performance of top 1 and top 2 classifications.

In fact, in many cases, it is difficult for a model to make a completely accurate decision. The greatest value of a model is that it can provide more suggestions for decision making. If the classification with the largest prediction probability is not accurate but the top 2 classifications are accurate, it could also provide considerable help for doctors. Here, we evaluated the precision of the 2 classifications with the largest prediction probabilities. In Figure 6, we can see that the precision of the first 2 categories of the model was 96.17% and there were few mistakes.

In addition, we explored the patient distribution according to average age and gender, as shown in Table 1. We observed that for FUO caused by tumors, the average age of patients was the highest at 50.91 years. Regarding FUO caused by immune diseases, the gender distribution showed a large difference, with 34.93% (803/2299) of patients being male and 65.07% (1496/2299) of patients being female. For the other 3 categories of FUO, there were no obvious differences in gender.

**Table 1:** Patient distribution in the data set.

	Patient demographics (N=2299)		
Categories of cause of fever of unknown origin	Mean age (years)	Male, n (%)	Female, n (%)
Infections	47.79	1226 (53.33)	1073 (46.67)
Immune diseases	42.90	803 (34.93)	1496 (65.07)
Tumors	50.91	1201 (52.24)	1098 (47.76)
Others	38.81	1268 (55.15)	1031 (44.85)

Usually, before doctors diagnose the cause of a patient’s FUO, they need to make an appointment to examine the patient. Table 2 shows the top 10 laboratory measurements that were associated with an FUO cause diagnosis. Percentage of basophils was the measurement that showed the strongest correlation with the FUO cause diagnosis. The other 9 measurements were percentage of large unstained cells, age, fibrinogen level, thrombin time, alkaline phosphatase level, direct bilirubin level, blood sodium level, 24-hour urine volume, and lymphocyte count. The top 10 measurements could be provided to doctors as laboratory appointment decision support.

**Table 2:** Top 10 laboratory measurements related to the diagnosis of the cause of fever of unknown origin.

Number	Top 10 laboratory measurements
1	Percentage of basophils
2	Percentage of large unstained cells
3	Age
4	Fibrinogen level
5	Thrombin time
6	Alkaline phosphatase level
7	Direct bilirubin level
8	Blood sodium level
9	24-hour urine volume
10	Lymphocyte count

## Discussion

### Principal Findings

Intelligent diagnosis of the cause category of FUO is significant and practical. With the rapid development of information technology, big data has been the focus of many fields in recent years [22]. Volume, variety, velocity, and value are the 4 “V” characteristics, although mining the deep value of data sets is the most important aspect for big data research. Similarly, in the medical field, large amounts of health care data are produced every day, such as EMRs, laboratory results, and images [23]. Considerable amounts of precious information can be extracted and mined from medical data using the proper methods. Previously, experts manually identified and analyzed the meaning of health data [24], which was time-consuming and difficult to identify specifically. Medical big data is increasingly more accepted by doctors because of its high efficiency and lower costs [25]. Rodger [26] mentioned that not only was a data extraction system needed, but medical big data applications such as those for clinical decision support were in urgent demand. Medical big data applications help make the medical process easier and friendlier for patients and relieve the pressure on clinicians. Among patients with FUO, the proportion of undiagnosed patients is approximately 20.5% [13]. In particular, the treatment for FUO may be much different, even contrary, for different causes. Therefore, helping doctors discover the specific cause as soon as possible is meaningful.

We addressed the problem using more appropriate and superior methods. Currently, medical big data applications have explored many directions [27,28]. The different kinds of methods used can be divided into 4 types: data mining, image recognition, natural language processing (NLP), and speech recognition. For example, intelligent diagnosis with the data excluding images [29,30] and intelligent early warnings [31] are both data mining problems. For image recognition [32], Simonyan and Zisserman [32] examined very deep convolutional networks and achieved superior performance. NLP is mainly used in the structured analysis of EMRs [19,33]. In this research, NLP also played an important role in structuring the text data, and we processed the EMR data using BERT and Jieba. Speech recognition [34], mainly addressed using recurrent neural networks, could help doctors transfer voice to text with high efficiency. Regarding medical big data models, there are unsupervised learning models and supervised learning models [35], such as logistic regression, decision tree, deep learning, and others. Currently, supervised learning models are used more often because of the sensitivity of the data to medical knowledge. As most problems in medical big data are classification problems, decision trees and deep learning models could achieve better performance. Wu et al [36] exploited the diagnosis of hypocellular myelodysplastic syndrome and aplastic anemia, and their experiments showed that the decision tree model outperformed the others in classification. Most importantly, deep learning methods require very large data sets [37], usually millions of data sets. Since there were only 2299 cases in this study, gradient boosting methods were better for this research. Therefore, LightGBM and XGBoost were used to train the data. In addition, 1372 cases not clearly diagnosed were removed from the study, and this kind of case would exist in the real-world setting. Therefore, the precision might be lower in reality, and these cases should be taken into account in future work.

### Conclusion

A machine learning method was innovatively introduced into FUO diagnosis. We presented the FUO intelligent diagnosis model called the FID, which was based on basic information, laboratory data, and EMR data from Peking Union Medical College Hospital. After cleaning the disordered data and structuring the text data using BERT, we conducted many experiments on the sample data and compared the performances from several angles. The results showed that the FID outperformed the comparative methods. As the treatments for FUO from different causes are very different, intelligently diagnosing an FUO into a category is meaningful. Our research was based on data from 1 hospital, and we intelligently diagnosed the FUO to 1 category of causes. In the future, we will focus on predicting the exact cause of an FUO using multicenter data. We would include all cases, including cases with no confirmed diagnosis, in our future research to better match real-world scenarios, which would probably improve the method more practical for the real clinical process.

## Abbreviations

BERT: bidirectional encoder representations from transformers
EMR: electronic medical record
FID: fever of unknown origin intelligent diagnosis
FUO: fever of unknown origin
NLP: natural language processing
PRISMA: Preferred Reporting Items for Systematic Reviews and Meta-Analyses
